# P-1876. Implementing a Transitions-of-Care Pilot for Patient Discharge on Outpatient Parenteral Antimicrobial Therapy (OPAT): Feasibility and Impact with Focus on Social Determinants of Health (SDOH)

**DOI:** 10.1093/ofid/ofae631.2037

**Published:** 2025-01-29

**Authors:** Colleen Burgoyne, Mike Sportiello, Sonal Munsiff, Alexandra Yamshchikov

**Affiliations:** Univ. of Rochester, Rochester, New York; University of Rochester Medical Center, Rochester, NY; University of Rochester, Rochester, NY; University of Rochester School of Medicine and Dentistry, Rochester, New York

## Abstract

**Background:**

Complex care coordination and requirement for rapid acquisition of advanced clinical skills at transition from inpatient settings to OPAT may lead to negative experiences and adverse outcomes for patients, with disproportionate impact on historically minoritized groups.

**Figure 1:**
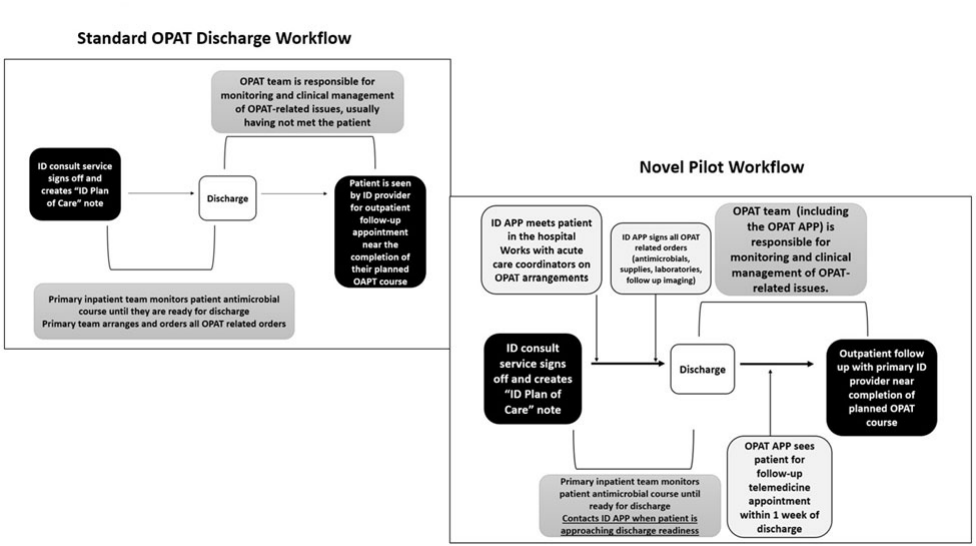
Implementation of a Novel Transitions of Care Pilot Workflow for Patient Discharges on OPAT Comparison of steps in previous discharge workflow to new process implemented for patient discharges on two pilot floors at URMC

**Methods:**

A novel workflow was implemented on two pilot units (Fig 1), using the OPAT Advanced Practice Practitioner (APP) to facilitate transition from inpatient to outpatient settings. Discrete OPAT APP practice changes included inpatient follow-up prior to discharge, completion of OPAT-relevant discharge orders, and telemedicine follow-up within one week post discharge. OPAT patients discharged 7/15-10/31/2023 from pilot units were included, with OPAT panel unaffected by pilot as comparator group. Patient records were reviewed for race and other socioeconomic parameters, comorbidities, OPAT-related discharge order errors, emergency department (ED) utilization, access complications, adverse events, and outcomes. A composite Undesirable OPAT Outcome measure was derived. Statistical analysis used chi-square, t-test in Microsoft Excel and Graphpad and logistic regression with R v4.3.3, rstatix v0.7.2, and sjPlotv2.8.15.

**Table 1.**
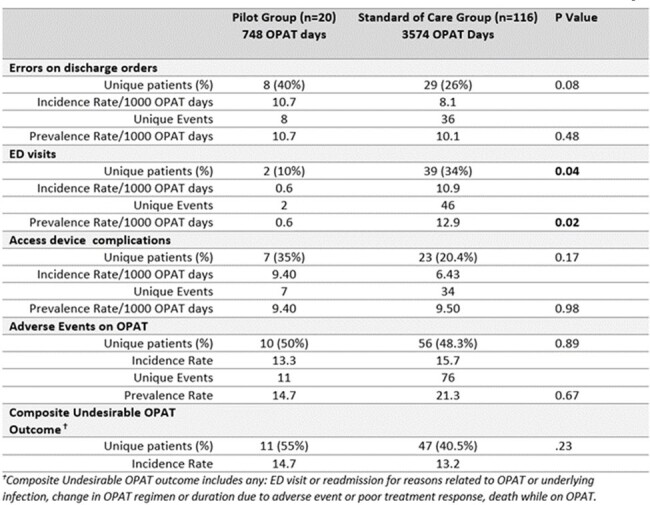
Comparison of Discharge Error Rates, ED utilization, Adverse Events and Treatment Outcomes between Pilot and Standard of Care Group P values with statistical significance are marked in bold

**Results:**

Pilot and comparator groups of 23 and 116 patients were similar in demographics, SDOH parameters, comorbidity, OPAT type, and antibiotic selection. Although rates of discharge order errors, adverse events, treatment outcomes were similarly high, pilot patients had lower rates of ED utilization (2.67 vs. 12.87/1000 OPAT days, p=0.02, OR 0.219) (Table 1), and shorter time to ID follow up (mean 5.1 vs. 23.8 days, p < 0.001), completing more follow-up appointments per course (2.25 vs. 0.9, p < 0.001). Despite variable penetration of the full intervention (48% of pilot group), 34 billable encounters were completed. In logistic regression and time-to-event analysis, pilot intervention may moderate effects of SDOH parameters on likelihood of order errors (Fig 2) and time to ID follow up (Fig 3).

**Figure 2.**
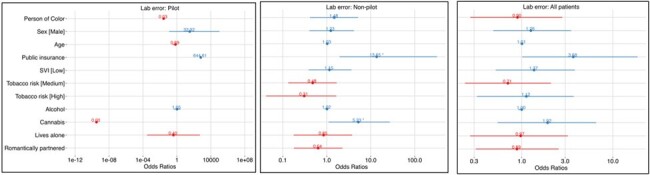
Odds Ratios of Laboratory Order Errors in Pilot and Standard of Care Subgroups by SDOH Variables

Univariate regression analysis of discharge order error outcome by SDOH parameters, odds ratios with statistical significance are marked with an asterisk

**Conclusion:**

Utilizing the OPAT APP in a boundary-spanning role focusing on transitions of care at discharge promotes improved follow up, ED utilization, and billing in OPAT-related care coordination, and may moderate impact of SDOH for minoritized patient populations receiving OPAT.

**Figure 3:**
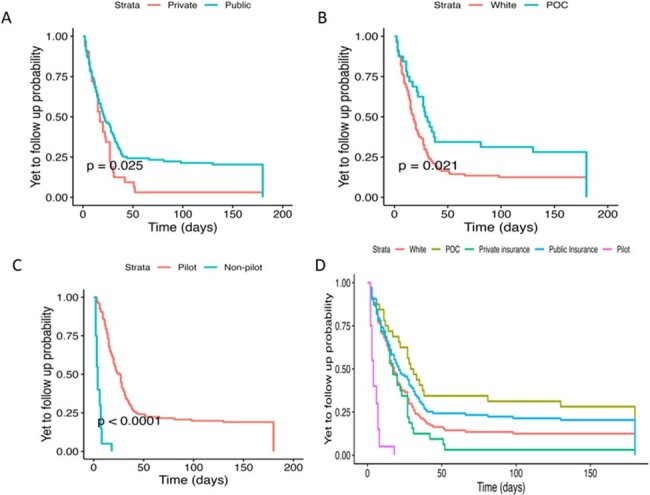
Time-to-event probability analysis for timing of post-discharge ID clinic follow up among OPAT patients Time-to-event curves for time (days) to ID follow up by Insurance Type (3A), Race (3B), Pilot and Standard of Care Status (3C), and All Patients (3D)

**Disclosures:**

All Authors: No reported disclosures

